# The Late Mr. William Sutton

**Published:** 1913-10-04

**Authors:** 


### THE LATE MR. WILLIAM SUTTON, J.P.

The Royal Victoria Infirmary, Newcastle-upon-Tyne, has suffered the loss of a valued worker and friend through the death of the late Mr. William Sutton, J.P. Mr. Sutton was a former Mayor of the City, and for twenty nine years an alderman, from which dignity he retired in 1908. As the head of an old-established business he was well known throughout all Newcastle and district, and was the chairman and director of several public companies in the North of England.

Mr. Sutton's association with the Newcastle Infirmary extended over many years. He took an active part, in co-operation with Sir Riley Lord, in raising over £100,000 for the building of the new infirmary in commemoration of the sixty years' reign of Queen Victoria. As a member of the house committee and chairman of the finance committee, he rendered most valuable service up to the time of his death. At the quarterly court of governors recently held reference was made to Mr. Sutton's long and helpful service to the charity, and a vote of sympathy was accorded to his widow and family.

